# An economic model of multi-level marketing

**DOI:** 10.1371/journal.pone.0253700

**Published:** 2021-07-20

**Authors:** Yaniv Reingewertz

**Affiliations:** Department of Public Administration and Policy, School of Political Sciences, University of Haifa, Haifa, Israel; University of Chester, UNITED KINGDOM

## Abstract

This paper offers an economic model of the operation of multi-level marketing (MLM) firms in competitive and non-competitive markets. The model takes a recursive approach to analyse decision making at the distributor level in order to understand basic issues in the MLM market and firm structure. Specifically, it is shown that under reasonable assumptions MLM firms will have a limited structure. In cases where commissions increase with the number of levels, MLM firms will include no more than six to nine levels in equilibrium. In cases of fixed commissions, market conditions dictate a cap on the number of distributors. These conditions imply a limited “multi-level” structure. They also imply that the revenues of the median distributor are mainly a result of direct sales and not a result of commissions. The model also suggests that MLM firms will only arise where marketing costs are substantial, and that it is primarily individuals with small outside offers who choose to become distributors. Finally, the model provides a formula that calculates market prices for a monopoly MLM firm.

## 1. Introduction

Multi-level marketing (MLM), also called network marketing (NM), is a business method used by some direct sales firms whereby individual distributors are encouraged to recruit new distributors. Distributors are paid a commission both on their own sales and on recruitment or sales made by their recruits, creating a multi-level marketing structure. This method became popular in the twentieth century, with companies like Amway, Avon, Herbalife and Nu Skin among the most well-known examples. Multi-level marketing is a multi-billion-dollar industry which amounts to roughly 1% of retail sales in the US [1]. A 2018 survey found that 7.7% of the US adult population had participated in at least one MLM organization during their lifetime [2]. This industry also manifests high growth rates in developing economies.

Multi-level marketing has garnered a considerable amount of criticism, partly on normative grounds [3–7]. Some of the criticism comes from the fact that some MLM firms have been determined to be operating as illegal pyramid schemes [1]. Another criticism stems from the use of allegedly unethical sales tactics by some of these firms (see, e.g., [4, 5]). At the same time, scholars suggest that MLM firms can operate without being defined as pyramid schemes. Possibly the biggest difference between a legal MLM firm and an illegal pyramid scheme is that legal MLM firms, unlike pyramid schemes, rely mostly on sales to consumers outside the MLM [1, 8, 9]. Note that the existence of some sales to consumers is not enough to protect MLM firms from pyramid scheme charges.

Multi-level marketing is a general term which describes a multitude of firm structures, all multi-level in nature. Each MLM firm has a unique compensation plan for its distributors, including various bonuses and commissions, which usually depend on the distributor’s level within the firm. These compensation plans can be classified based on two criteria. The first is the way commissions are paid, and specifically, whether commissions (or bonuses) are paid based on sales or recruitment [1]. The model presented in this paper differentiates between these two types of commissions, thus allowing for a general discussion of MLM firms. The second criterion which differentiates between MLM firms is the existence of horizontal and vertical restrictions on the multi-level structure. These restrictions limit the number of distributors in each level (horizontal restrictions) or the number of levels (vertical restrictions). By doing so they create four general plan structures: uni-level, binary, matrix, and breakaway, where the uni-level and binary plans are the most common types [10]. The uni-level plan imposes no restrictions on the number of distributors in each level, and no restrictions on the number of levels, while the binary plan allows for only two distributors in each level [11–13]. The matrix plan typically restricts the number of distributors in each level to between 2 and 5, while also limiting the number of levels [10]. Finally, the breakaway plan ensures restrictions on the number of levels by disconnecting distributors from their upline distributors once they reach a certain network size. The model presented in this paper imposes no restrictions on the number of distributors, and thus represents the uni-level plan. This is done in order to allow as much flexibility and generality as possible. The model can be extended to allow for restrictions on both the number of levels and the number of distributors in each level.

Despite the size of the MLM industry and the complexity of its business model, multi-level marketing is under-studied in both economics and marketing. In particular, there have been very few attempts to provide an analytical framework to discuss the way MLM firms operate. Such an analytical framework is needed if one wishes to understand the business activity of MLM firms and whether regulatory intervention is needed. The purpose of this paper is to offer such an analytical framework, via an economic model which takes a recursive approach to analyse the optimizing behaviour of a potential MLM distributor. The model helps answer several key questions relating to the design and implementation of MLM–specifically, how many levels an MLM firm will include and whether promises of an almost-infinite multi-level structure are warranted; why MLMs are present in some markets but not in others; how the fee structure of MLM firms is being determined; and why low-skilled workers tend to join MLM companies. Finally, I analyse the case of a monopoly MLM and show how market power affects prices in an MLM market.

The main contribution of this paper is to explain and model the operation of MLM firms from an economic standpoint. Specifically, I take a recursive approach which connects market fundamentals at the lowest-distributor level to the operation of the entire MLM system. This approach does not require assumptions about diffusion processes and does not model network structure, other than the number of levels.

The main finding suggests that MLM firms will tend to have very few levels. Indeed, under reasonable assumptions the number of levels will not exceed nine, and probably even six. Under such conditions the earning potential of the typical distributor from recruiting further distributors is quite limited. This proposition is consistent with the findings of the few empirical studies found in the literature [14–16]. The model also suggests that marketing costs dictate the ability of MLM firms to operate and expand. In competitive markets, the fees paid to MLM distributors depend on marketing costs borne by non-MLM firms. This puts a cap on both the number of levels within the MLM firm and the fees it pays its distributors. The model also explains which populations are more likely to be attracted to this business opportunity, and suggests that these mainly consist of individuals with a relatively small earning potential. This finding helps explain the rapid penetration of MLM firms to markets in developing countries. Finally, the paper discusses the case of a monopoly MLM and shows that while it offers a questionable business opportunity for distributors, it poses less of a threat to consumer welfare compared to standard monopolies.

## 2. Related literature

Within the marketing and economics literatures, only a few papers deal with issues directly related to multi-level marketing firms. One of the key questions which was explored is the difference between legal MLM firms and (illegal) pyramid schemes. [1] suggest a key difference between MLMs and pyramid schemes, and show that MLMs are based on sales to consumers outside the firm while pyramid schemes rely mostly on purchases within the pyramid structure. Still, in real life some pyramid schemes disguise themselves as MLM firms [16]. [17] shows that non-rational agents are willing to participate in pyramid schemes and also discusses differences between MLM firms and pyramid schemes. Another related paper [18], deals with the relationship between MLM levels, focusing on behavioural or social interactions and how they are translated into sales.

Several papers from the exact sciences provide insights into the operation of MLM network structures. [13] offer a physics model for the operation of MLM firms, and show that network growth is not unlimited. [19] and [20] use network theory to describe how MLM firms evolve through time and the structure they take. Finally, [12] take an axiomatic approach to study different reward mechanisms available to MLM firms. They suggest that geometric rewards mechanisms satisfy several desired properties of MLM networks. The economics literature has also addressed the more general issue of network structures. see, e.g., [21–23]. The limitation of taking a physical network approach is that it does not deal with economic incentives and market structure. This is why [13] find that the number of levels in the MLM firm can be as high as 30.

This paper is most closely related to [24], who provide a model of MLM firms which captures several attributes of this market. Their focus is on the firm level and they do not model market and firm structure and market equilibrium. In addition, they do not try to estimate the number of levels in the MLM firm. I contribute to this literature by providing an economic model which is recursive in nature. The model focuses on multi-level marketing firms, analyses the incentives faced by the distributor, and provides a competitive equilibrium outcome using a recursive approach which best fits the MLM structure. The model allows us to answer ongoing questions in MLM firm and market structure.

Most of the literature on MLM firms is theoretical in nature, and the empirical literature on these firms is very thin. One strand of this literature shows that MLM distributors tend to come from low socio-economic backgrounds [25]. For example, [15] survey a sample of lowest-level distributors in a Japanese MLM firm. They find that 90% of these distributors were women who worked part-time in MLM activity. [26] also address descriptive features of MLM participants; they show that demographic homophily influences network structure (see also [27]). [28] find that the highest penetration rates were achieved in counties comprised of large affinity groups such as religious communities and Hispanic populations. The second strand of the empirical literature documents the low earnings of most distributors in the MLM industry [29]. [16] report that the average yearly earnings of distributors in two big MLM firms are about $700. [14] analyse the case of FHTM–an MLM firm which was found to be operating as a pyramid scheme in Montana. They analyse the diffusion processes of the firm and suggest that the maximum penetration was 1% of the population, and that 94% of participants did not make profits. [30] provide similar results, suggesting that only 6.5% of distributors earn commissions on recruitment. [31] suggest that roughly 50% of distributors lose money, roughly a quarter break even, and another quarter make some profit. [25] analyses three MLM firms and concludes that most distributors do not earn profits.

This paper is also related to three other strands of the literature. First is the literature on consumer referrals [31, 32]. This literature is also related to the issue of consumer rebates (see, e.g., [33]). In fact, commission fees in multi-level firms can be thought of as paying customers for consumer referrals. The main difference is the multi-level structure, which is not present in consumer referrals. In addition, MLM firms offer a business opportunity, whereas consumer referral bonuses are usually more limited in scope. Second, the paper has some links to the literature on firm hierarchy. Multi-level marketing creates a hierarchical structure with potentially many levels, at least in the marketing activity of the firm. [34] shows how a specialized acquisition of knowledge creates hierarchies, where production workers solve the easy tasks and upper-level management deals with more complicated tasks (See also [35] for empirical findings on firm hierarchies). This model is consistent with the standard MLM structure, where distributors take the place of production workers. [36] suggest that labour-intensive industries will include firms with flatter hierarchies than those in capital-intensive industries, because workers can open their own firm and compete with the existing firm. Moreover, [37] report that hierarchies are in fact becoming flatter over time. While MLM firms, which supposedly have steeper hierarchies, would seem to be an exception to this finding, the model which is presented in this paper suggests that this is not necessarily the case.

Finally, the paper is also related to the principal-agent literature, since the incentive structure of MLM firms essentially entails a contract between a principal (the upper-level distributor) and an agent (his downline distributor). [38, 39] consider the delegation of power and responsibilities within the firm through a principal-agent framework. They suggest that in cases where agents have an informational advantage over their principals, there will be greater decentralization. This is potentially the case in MLM firms where distributors supposedly have more knowledge regarding their marketing activity. While the principal-agent framework is very useful for the study of MLM firms, the specific structure of these firms is not explored in this literature. Nor, to the best of my knowledge, has there been any discussion regarding how the principal-agent model may be used to explore MLM network structure.

## 3. The model

### 3.1 MLM in competitive markets

To understand the structure of MLM firms, let us lay out a simple economic model of the operation of a multi-level marketing firm. I will focus on the distributor, and examine how distributors operate depending on their level within the organization. For simplicity and generality, I will assume a uni-level structure. The analysis can be extended to other structures, such as the binary and matrix types. In both the binary and matrix plans there are more constraints on the multi-level structure of the firm, resulting in smaller firm sizes compared to the uni-level case. In this regard our model allows for more flexibility, more levels, and more distributors in each level. I assume that the distributor must divide his time between two different work activities: marketing the product to potential customers, and recruiting new distributors (e.g., through seminars, home visits and conventions). I assume also that the distributor can engage in only one of those activities–recruitment or sales–at any given time. I later relax this assumption (see Section 3.3.2). Let *t* denote the fraction of time he devotes to sales, and *1-t* the fraction of time he devotes to recruitment.

Assuming separability between sales and recruitment may seem a bit strong at first glance, since in real life the efforts of a distributor to sell the product may bring him new recruits, and vice versa. However, this is a simplifying assumption which doesn’t affect the main results of the model. This is because in the model the activity of the MLM structure depends on the behaviour of the lowest-level distributors. The lowest-level distributors, by definition, do not engage in recruitment. Being a lowest-level distributor means that you will not be able to recruit distributors, and since people in our model are rational these distributors will not engage in futile activity. Therefore, the influence of sales on recruitment and vice versa does not affect the behaviour of the lowest-level distributors.

The assumption of separability between sales and recruitment also makes sense since the two activities are directed to two different segments, where the first are people interested in buying and using the product, and the second are people looking for a business opportunity. It is worth noting that the model deals with legal MLM firms which do not rely on sales to distributors. Indeed, many MLM firms expect new recruits to purchase the product for their own use. However, if these recruits are not really interested in using the product than these purchases just mean they are left with lower profits.

Sales by the distributor are denoted by *Q*_*i*_ and are a function of time spent on sales–*t*. Sales of each downline distributor *j* are denoted by *Q*_*j*_. The existence of distributor *j* depends on 1−*t*_*i*_ of distributor *i* (1-t_i is the time distributor i dedicate to recruitment), since without the recruiting efforts of distributor *i*, there will be no distributor *j* and *Q*_*j*_ will be zero.

Subscript *i* denotes the level of the distributor, which takes the values zero (for the upper-most distributor) to I (total number of levels, also the level number of the lowest-level distributor). Distributors in the same level have the same incentives and constraints. I note here that the model does not impose conditions on the number of distributors in each level, and does not focus on this issue. As noted above, the model takes a recursive approach and deals with the lowest-level distributors, which affect all other levels. Assuming perfect competition means that the price is fixed hence the number of distributors in each level does not affect the price. I assume *Q*_*i*_ is concave with respect to t ($\frac{dQ}{dt}>0,\frac{d^{2}Q}{dt^{2}}<0$), and that *Q*_*j*_ is concave with respect to *1-t*.

The distributor sells the product at the market price, *P*, and pays the MLM firm a cost of *C* (the wholesale price). I assume that the market for the product is perfectly competitive. In competitive markets the demand faced by each distributor is unlimited at price *P*. I will later deal with non-competitive markets where distributors can affect the price. Finally, the distributor receives a commission or fee, *α*, on sales made by his downline distributors. Equally, the distributor must pay a similar fee on his own sales to his ‘upline’ distributors. In some cases fees come in the form of a higher wholesale price, but the principle remains the same. A change in how I model fees does not affect the results. The exact structure of these fees is described below.

The earnings of a distributor at level *i*, denoted by *π*_*i*_, are given in Eq 1:

$$\pi_{i}=[P-C-i^{\beta }\alpha ]Q_{i}(t)+\sum_{j=i+1}^{I}{\frac{\alpha }{j^{\beta }}Q}_{j}\left(t_{j,}1-t\right)$$ (1)

The first right-hand-side term represents the distributor’s profits from his own sales, calculated by subtracting the wholesale price, *C*, and fees to up-line distributors, *i*^*β*^*α*, from the market price, *P*, and then multiplying that result by the quantity sold.

I allow for a general structure of the commissions, depicted by *i*^*β*^*α*. To clarify this notation, let us take two cases, which are described and analysed below (Sections 3.1.1 and 3.1.2). In the first case beta equals one, hence the distributor must pay a fee of *α* to each distributor up-line from him. This is a simplifying assumption which also holds true in some MLM firms. In the second case beta equals zero, hence the distributor needs to pay only a fee of *α* for each unit sold. To illustrate the differences between these two cases let us take an example of a distributor who has two distributors above him. In the first case he would need to pay 2*αQ* and in the second case he would only need to pay *αQ*. This difference in the structure of commissions dictates stark differences in the structure of the MLM network, as described in Sections 3.1.1 and 3.1.2 below.

The second term on the right-hand side represents the distributor’s profits from recruiting downline distributors, namely the sum of the fees paid to him by each downline distributor. This term also depends on beta and the number of levels above the distributor. For simplicity and tractability I assume separability of *Q*_*i*_ and *Q*_*j*_—that is, time devoted to sales does not help in recruiting downline distributors, and vice-versa. This assumption simplifies the model and is plausible if the business opportunity of becoming an MLM distributor is not linked to the consumption of the products of the firm. This is arguably the case in legal MLM firms which do not rely on sales within the MLM network [2].

#### 3.1.1 MLM with increasing commissions

The general formula for the distributor’s profits is given by Eq 1, where the parameter beta allows different commission structures. For the sake of concreteness, I assume here that beta equals one. This creates a commission structure which depends on the level of the distributor. Each distributor pays a commission of *α* to each of the distributors above him. Thus, a distributor at level *i* pays commissions of *iαQ*. This assumption more closely matches some MLM firms, but admittedly not others. This new commission structure is given in Eq 1A below, which is a variant of Eq 1:

$$\pi_{i}=[P-C-i\alpha ]Q_{i}(t)+\alpha \sum_{j=i+1}^{I}Q_{j}(t_{j,}1-t)$$ (1A)

Where the distributor’s earnings denoted by *π*_*i*_ are a function of direct sales (first right-hand-side term) and commissions from downline distributors (second right-hand-side term).

Previous studies (e.g. [20, 24]) modelled the MLM firm as having a potential to reach an infinite number of levels. This assumption, while appealing, ignores the dependency of upper-levels distributors on the business activity of lower-levels distributors. These interrelations, which are the result of marketing commissions paid by downline distributors to upline distributors, dictate a hierarchical structure and therefore call for a recursive approach. Under this approach the earnings of each distributor depend on the earnings of the lowest-level distributor, via the fees paid to upline distributors. This dependency means that the decisions of each distributor depend on the decisions of downline distributors. This is why a recursive approach is needed.

Due to the hierarchical structure described above I assume a finite number of levels (See S1 Appendix for a model with an infinite number of levels). To analyse this model I take a recursive approach, starting from the distributor at the lowest level. This distributor joins the MLM firm if he can make earnings which are equal or greater than his outside offer. This condition creates a restriction on the number of levels and therefore affects all his upper-levels distributors.

Let us look at the lowest-level distributor, who by definition is unable to recruit additional distributors. He has the following earnings function (taken from Eq 1A, but without earnings accruing from downline distributors):

$$\pi_{i}=[P-C-I\alpha ]Q_{i}(t)$$ (2)

I continue to assume that the product is sold in a perfectly competitive market; i.e., that its price is fixed. This means that the price is determined by the market and not by the firm. Let us assume that this competitive price is equal to *C+m*, where *m* is the unit marketing cost of non-MLM firms, and *C* is the unit production cost (including managerial costs and financing), which is assumed to equal the wholesale price of the product for the MLM firm. Notice that the price in the market is dictated by production and marketing costs of non-MLM firms. This is a plausible assumption because if marketing costs of MLM firms are higher than those of non-MLM firms they will need to charge a higher price than non-MLM firms to recover these costs and would therefore be unable to compete in the market. Therefore, in competitive markets, marketing costs of MLM firms are bounded by the marketing costs of non-MLM firms.

To deduce the behaviour of the lowest-level distributor, I equate the earnings of this distributor, equal to [*m*−*Iα*]*Q*_*i*_(*t*), to what he would have earned through his outside option (I take the earnings depicted in Eq 2 and substitute P = C+m). The outside option is assumed to be either a full-time position in a non-MLM firm or self-employment, and is denoted by *wL* (*w* for hourly wage, *L* for hours of work). An equation between earnings through multi-level marketing and the outside option is a condition for equilibrium in the labour market in the presence of an MLM firm, and is depicted (after rearrangement) in Eq 3:

$$I=\frac{\left[m-\frac{wL}{Q}\right]}{\alpha }$$ (3)

Eq 3 provides a formula to calculate *I*–the number of levels of the MLM firm. The number of levels is positively associated with marketing costs, but negatively associated with the commission fee and the outside option. In fact, marketing costs dictate the number of levels in equilibrium, conditional on commission fees. This is the first interesting result of the model, which implies that MLM firms will be larger (i.e., will include more levels) in markets with higher marketing costs. This is the case because the MLM firm has to compete with standard firms, and having more levels means a smaller margin for the downline distributor.

Another interesting result which stems from Eq 3 is the negative link between the size of the commission fees and the number of levels. Having larger fees means that the earnings of the low-level distributors are lower, and they essentially allow for less levels in the MLM structure.

It is important to elaborate regarding the link I find between commission fees and the number of levels (or the number of distributors in the second version of the model; see Section 3.1.2). While some may consider this finding trivial, there are several reasons it should be noted. First, previous literature has failed to acknowledge this link (see, e.g. [24]). Second, it is important to be able to offer specific estimates of the number of levels MLM firms will have, especially given the elevated earlier estimates for the number of levels in the literature (see, e.g., [13]). Finally, from an applied perspective, there is value to correcting the common wisdom in the public and policy arenas, where this link has not previously been recognized.

Now that we know the number of levels, let us see how a distributor at level *I-1* –that is, the second-lowest level–decides on his time allocation. He will maximize the following condition:

$$\pi_{I-1}=[P-C-I\alpha ]Q_{i}(t)+\alpha [Q_{I}(1-t)]$$ (4)

Which yields the following first-order condition:

$$[P-C-I\alpha ]\left(\frac{dQ_{I-1}}{dt}\right)=\alpha \left(\frac{dQ_{I}}{dt}\right)$$ (5)

I assume a Cobb-Douglas production function where *Q*_*i*_ = *t*^0.5^ (and, respectively, *Q*_*j*_ = (1−*t*)^0.5^). The Cobb-Douglas production function is probably the most commonly used production function in economics, and has been found to accurately describe production conditions in many industries [40–42]. I then substitute *P = m+C*, *m* = *Iα*+*wL*/*Q* (from Eq 3) and maximize Eq 4 for the distributor at level I-1. This produces the following equation:

tI−1=(wLQ)2α2+(wLQ)2 (6)

That is, a distributor at the second-lowest level dedicates more time to direct sales, *t*, and less time to recruitment when his outside offer is larger. On the other hand, he dedicates less time to sales and more to recruitment when fees are larger.

Performing the same maximization for distributor *I-2*, I get

tI−2=(wLQ)24α2+(wLQ)2 (7)

Recursively, the general formula is:

tI−j=[wLQ]2(I−j)2α2+[wLQ]2 (8)

In other words, time spent on sales, *t*, declines as one moves up the levels (for example see Eq 7 vs. Eq 6, where the denominator includes the term 4*α*^2^ instead of *α*^2^), so that distributors at the top levels devote more time to recruitment compared with their downline distributors. This result is the opposite of the result obtained for the non-recursive model (see S1 Appendix). Time spent on recruitment is negatively associated with the outside offer, and fees negatively affect time spent on sales (and increase time spent on recruitment).

*3*.*1*.*1*.*1 Comparative statics*. I can demonstrate the importance of marketing costs through an example using Eq 3 and parameter values taken from the US. Assume that the outside offer, *wL*, equals the minimum wage in the US ($7.25 per hour or $330 for a 44-hour work week). Assuming an outside offer which equals the minimum wage is consistent with the empirical observation that most MLM distributors work part-time [15, 16]. I take the number of average weekly working hours from the BLS, and *Q* equal to $10,000. It is difficult to estimate a reasonable value for Q, which is the weekly quantity sold by one distributor with t = 1. This quantity depends on the distributor’s marketing ability and the level of competition in the market. Therefore, our choice of Q = 10,000 is somewhat arbitrary. Nevertheless, since in reality most distributors work part-time and don’t earn much [15, 16], it is probably a fairly large number, thus conservative from our point of view (i.e. it will result in an upper-bound estimate for the number of levels).

Table 1 presents the number of levels under various parameter values. I explore three magnitudes of marketing cost margin (10%, 20% and 30%), and two different commission fees (5% and 3%). Commission fees of 5% are the case in some MLM firms, e.g. Herbalife. Tupperware offers commissions which range from 4% to 8% for the manager level. As can be seen in the table, the model predicts that MLM firms will have a very limited “multi-level” structure with a 10% marketing cost, having one or two levels. Under a 5% fee the multi-level firm has no more than six levels, and with a 3% fee the maximum number of levels is nine. I note that our estimates are an upper bound for the actual number of levels in such cases. This is because our estimate for sales (*Q*) is large and that of the outside option (*wL*) is low. Choosing a larger outside option or a lower level of sales will reduce the number of levels. These striking results suggest that MLM firms will have a relatively modest number of levels in equilibrium, and that distributors will derive most of their revenues from direct sales, as there will be few downline distributors below them.

**Table 1:** Comparative statics.

	m	*α*	$\frac{wL}{Q}$	Number of levels
1.	10%	5%	0.033	1
2.	20%	5%	0.033	3
3.	30%	5%	0.033	5
4.	10%	3%	0.033	2
5.	20%	3%	0.033	6
6.	30%	3%	0.033	9

Note: m is marketing cost as a percentage of the total cost. *α* is the commission fee. $\frac{wL}{Q}$ is the outside offer divided by revenue from quantity sold. The number of levels is rounded.

In our model, which focuses on the distributor level, commission fees are taken as exogenous, as they are set by the MLM firm. Nevertheless, Eq 3 can hint at the size of these fees: in order to increase profits, the MLM firm may want to lure more distributors by increasing fees, but it may also want to increase the number of levels in the firm, which means lower fees (since both are capped by marketing costs). In the example given above (with an outside offer the size of the minimum wage), if fees are 10%, the number of levels is very small. This implies that commission fees are essentially capped by the marketing margin. This result helps to explain the size of commission fees in MLM firms.

As mentioned above, Eq 3 can also help explain why MLM firms are usually found in markets that entail high marketing costs, such as beauty and wellness [16]. These large marketing costs allow for more levels, which is the prime condition for a successful MLM firm. Eq 3 can also explain the rapid penetration rates that MLM firms achieve in developing countries. Since the outside offer is negatively related to the number of levels in the firm, our model predicts that MLM firms will have more levels in poor countries, where the outside offer (i.e., a salary in a non-MLM firm) is fairly small. This also explains why many distributors are poorly educated or come from low socio-economic backgrounds, conditions that are associated with a lower outside offer.

*3*.*1*.*1*.*2 A motivating example*. To provide an example which will illustrate the applicability of the model, I pick the case of Fortune High Tech Marketing (FHTM) in Montana [14, 43]. I will focus on the issue of the number of levels that this MLM company had, and discuss the connection between this example and our model.

FHTM was founded in 2001 and was officially closed in 2014. Our analysis is based on its operation in Montana during the years 2006–2010. FHTM is a rare case where a rich dataset exists which describes the number of distributors, net earnings by distributor and other data which can help calculate the number of levels within the MLM firm. This rich dataset is a result of legal proceedings which found FHTM to be an MLM firm and a pyramid scheme and resulted in the shutdown of the firm. While this firm was found to be an illegal pyramid scheme, I believe we can still learn something from its multi-level structure. Data from this lawsuit is analysed by [14, 43], providing several key numbers and insights:

- The total number of distributors in Montana was 3,737, but only 1,689 were able to recruit downline distributors, and only 223 had net earnings above zero.
- Each distributor was allowed to recruit three downline distributors directly below him. Other recruits would form another level below him.
- Only distributors who recruited three downline distributors qualified for bonuses (these were termed Qualified Representatives, or QR). Therefore, distributors in the lowest level did not qualify for bonuses.
- The maximum net earnings for the most successful distributor amounted to $240,500.
- The FHTM business opportunity gave much emphasis to recruitment, as most commissions were recruitment-based.

According to [43], having five levels of qualified distributors (or six levels overall) will allow the highest-level distributor to receive $125,610 in recruitment bonuses alone. Having six levels of QR will allow him to receive $406,275 in recruitment bonuses (see Vander-Nat 2013, Table 6 and Table D5 in Appendix 2). Thus, since the actual net earnings of the person at the highest level were 240.5 thousand dollars (less than the earnings of the highest-level distributor when there are six levels), we can conclude that FHTM had five levels of QR while operating in Montana, or six levels if we include distributors who were not qualified to receive bonuses.

Another way of reaching the same conclusion is by comparing the number of distributors receiving positive net earnings (223 distributors) to the potential number of distributors in an MLM firm where each distributor recruits exactly three downlines. These numbers are 121 distributors for five levels and 364 distributors for six levels. Assuming that distributors who had zero net earnings or below were not able to have downlines, I reach again five levels of Qualified Representatives, or overall six levels.

Finally, note that the FHTM case may not be representative of the MLM industry, since it was declared a pyramid scheme, and since its rewards to distributors were mainly recruitment-based. Nevertheless, if MLM firms such as FHTM which reward recruitment to a large extent create a network structure of no more than six levels, it is hard to believe that MLM firms which focus less on recruitment will be able to form larger networks with more levels.

#### 3.1.2 MLM with fixed commissions

One of the key assumptions of the model described in Section 3.1.1 is that commissions increase as the distributor falls further down the MLM structure. For example, a distributor in level 5 will pay commission fees of 5*αQ* and a distributor in level 6 will pay 6*αQ*. While some MLM firms operate in such a way or using a similar scheme, others do not. MLM firms can have different schemes, and may not impose higher commission rates on lower-level distributors.

To more closely model such firms, the model is modified so that the commission rate is constant across levels. This is depicted in Eq 9, which is a variant of Eq 1, where beta equals zero:

$$\pi_{i}=[P-C-\alpha ]Q_{i}(t)+\sum_{j=i+1}^{I}{\frac{\alpha }{j}Q}_{j}(t_{j,}1-t)$$ (9)

Having beta equal zero has two important implications: first, the commission paid by the distributor to his upper-levels recruiters is independent of the level the distributor is in. Second, the distributor’s revenues from downline distributors faze-out the larger the distance between him and the downline distributor.

I continue to focus on the lowest-level distributor, who has no downline distributors. This distributor has the following entry condition, which compares his outside offer to his earnings in the MLM firm:

$$(P-C-\alpha )Q_{i}(t)\geq wL$$ (10)

Equating the outside offer to the MLM earnings, which is the equilibrium condition in this market, gives the following condition:

$$Q^{*}=\frac{WL}{P-C-\alpha }$$ (11)

First, notice that the condition described in Eq 11 does not impose any direct restrictions on the number of levels. This means that MLM firms which have commission rates which are independent of the level can have a large number of levels. However, this condition imposes a restriction on the quantity that the distributor needs to sell in order to stay in business. In this version of the model only people who are able to sell at least Q* will accept the MLM opportunity. This condition restricts the size of the MLM since quantity sold depends both on the demand for the product and on the ability of each distributor to market and sell the product. Note also that the quantity sold may depend on the level of the distributor, as low-level distributors may find it harder to find new customers. Taking this into account will imply having a more limited MLM structure.

I take two specific examples in order to illustrate this point, using real-life data. The first example uses Herbalife, one of the biggest MLM firms in the world. Herbalife’s most-sold product–its Formula 1 shake–is priced at roughly $38 (on eBay, retrieved August 4, 2019). According to Herbalife’s documents (the compensation plan), a distributor can expect a $9 profit for each product sold. If we plug this number into Eq 11 (instead of *P*−*C*−*α*), and assuming W = 7.5 and L = 194, we get Q* = 162, and monthly revenues (Q*P) of $6,156. I use the federal minimum wage ($7.5) and average work-week of 44 hours a week (194 hours a month) based on estimates by the Bureau of Labor Statistics. In other words, the condition described in Eq 11 implies that a full-time distributor of Hebalife must sell products worth at least $6,156, making a monthly profit of at least $1,458, if he is to find participation in the firm profitable. This condition sets a maximum for the market share of Herbalife, since distributors who are unable to sell at least 162 units a month will not remain active. Hence, under this version of the model the limit to the MLM growth is not described by a cap on the number of levels, but rather by a cap on the number of distributors who will find it beneficial to pursue this business opportunity.

One can take another illustrative example from Tupperware, another MLM firm. Tupperware’s most-sold product is the Wonderlier bowl set, priced at $35 (retrieved from Tupperware.com, August 4, 2019). According to the firm, distributors can expect a profit of 25%, or $8.75 in our case (Source: order.tupperware.com/coe-pdf/oppkit-0914.pdf). Plugging these numbers into Eq 11 gives very similar results to the Herbalife case: Q* = 166, revenues of $5,810 and minimal (full-time) distributor’s profits of $1,453. The similarity between these two examples (profits of $1,458 for Herbalife and $1,453 for Tupperware) is striking, but not surprising–both firms need to recruit (potentially similar) distributors and therefore need to offer similar profits.

### 3.2 Extensions

#### 3.2.1 MLM firms with asymmetric information

In the previous section 1 assumed that individuals have symmetric information. Specifically, I assumed that individuals know their level in the MLM structure, and hence their earnings potential. Thus, lowest-level distributors know that they will not be able to recruit additional distributors and focus on sales rather than recruitment. This assumption is in line with the findings of a lab experiment which showed that providing individuals with more information on potential earnings does not change the appeal of the MLM business opportunity [44]. However, assuming symmetric information may not be realistic in real-life scenarios, where the number of levels is not common knowledge [45]. Moreover, some MLM firms, or more exactly some distributors, suggest to new recruits that they will be able to recruit many downlines below them, regardless of the current number of levels in the MLM. The issue of asymmetric information is tightly linked to the assumption of rationality. Rational agents working under asymmetric information will form accurate expectations regarding their ability to recruit downline distributors. However, if individuals are not rational, they might be more susceptible to these promises.

In this section 1 will allow for asymmetric information and behaviour which implies bounded rationality. I will assume that lowest-level distributors believe they have the ability to recruit downline distributors. Hence, their profit equation is identical to that described in Eq 1A. I analyse here the case with increasing commissions (Section 3.1.1). Analysing the case with constant commissions (Section 3.1.2) gives similar results–asymmetric information pushes more people to become distributors, and they earn lower profits compared to the case of symmetric information (results available upon request). In order to derive the condition for equilibrium in this market I will proceed in two stages. First, the lowest-level distributor will maximize Eq 1 –to decide how to divide his time between recruitment and sales. This maximization yields t*, which is the (thought to be) optimal share of time devoted to sales. Note that t* is thought to be optimal by the distributor, but is not optimal since the (lowest-level) distributor is in fact unable to recruit downline distributors. In the second stage, this distributor checks whether his earnings as a distributor are greater than the outside offer (*wL*). Thus, the condition for equilibrium in this market is depicted in Eq 12:

$$\pi_{i}=[P-C-i\alpha ]Q_{i}(t^{*})+\alpha E[\sum_{j=i+1}^{I}Q_{j}(t_{j,}1-t)]=wL$$ (12)

Rearranging Eq 12 and plugging in *P = c+m*, gives the following condition:

$$I=\frac{\left[m-\frac{wL}{Q}\right]}{\alpha }+\theta$$ (13)

Where $\theta =\frac{E[\sum_{j=i+1}^{I}Q_{j}(t_{j,}1-t)]}{Q}$. Eq 13 is very similar to Eq 3, with *θ* being the only difference. This parameter captures the effect of asymmetric information, or bounded-rationality, on the number of levels. It is larger if the expected sales of downline-distributors are greater than the actual sales by the distributor. In other words, higher (irrational) expectations by lowest-level distributors will yield more levels in the MLM structure.

When distributors have non-rational expectations regarding recruitment success they are more likely to engage in recruitment. These additional recruitment efforts are not optimal from the point of view of the distributor, since they come at the expense of time devoted to sales, and they do not bring sufficient earnings accruing from fees from downline distributors. For example, the lowest-level distributor will engage in recruitment despite his inability to recruit downline distributors. This leads to more recruitment efforts and more levels. However, it has another effect which is to reduce earnings by the entire MLM structure, since distributors are over-recruiting. Thus, while getting an MLM structure with more levels, it is also a structure with smaller earnings for each distributor. Finally, as mentioned above, this structure characterises an equilibrium with individuals with asymmetric information. The existence of such an equilibrium in the long-run means that these individuals acquire no information as time goes by. However, if their recruitment efforts continue to be unsuccessful, it seems unlikely that they will not learn from this information. Hence, the persistence of these recruitment efforts require a substantial deviation from rationality which is a very strong assumption.

#### 3.2.2 Incorporating risk into the model

In the previous parts of the model, I assumed that as a business opportunity, becoming a distributor is not riskier than the outside option. However, distributors are usually self-employed, and being self-employed could be considered riskier than being a salaried worker. Hence, becoming an MLM distributor is potentially riskier than the outside option. Thus, it is worthwhile to explore whether incorporating risk into the model changes its results.

The simplest way to incorporate risk into the model is to add it to the entry condition of becoming a distributor. This condition equates the earnings of the distributor to the outside option. If becoming a distributor is riskier than the outside option then people will demand higher earnings as distributors compared to the outside option, so that they will be compensated for the excess risk. This risk premium is denoted by R and the full entry condition is described in Eq 14:

$$\pi_{i}=[P-C-i\alpha ]Q_{i}(t)\geq wL+R$$ (14)

where the earnings of the distributor are similar to Eq 2. I analyse here the case with increasing commissions (Section 3.1.1). Analysing the case with constant commissions (Section 3.1.2) gives similar results–the risk premium pushes Q* up, and fewer distributors will enter the MLM firm in equilibrium. These earnings now need to be higher or equal to the sum of the earnings of the outside option (*wL*) and the risk premium R. In equilibrium the right- and left-hand sides of Eq 14 are equated. After plugging in the marketing costs (*P*−*C* = *m*) and looking at the lowest-level distributor, I get Eq 15:

$$I=\frac{\left[mQ-wL-R\right]}{\alpha Q}$$ (15)

As can be seen in Eq 15, the number of levels goes down with the risk premium. In other words, incorporating risk into the model leads to having fewer levels in equilibrium. The effect of the other parameters of the model on the number of levels is similar to the previous results of the model.

### 3.3 A monopoly MLM firm

Until now I have assumed that the MLM firm operates in a competitive market. However, some MLM firms claim to have a unique or innovative product, making the case of a monopoly potentially more relevant [16]. [46] find that MLM distributors in Malaysia do not perceive MLM products to be more innovative than products of competing non-MLM firms. In addition, even if the product itself is not a monopoly, the business opportunity the MLM firm is offering may be considered as such, at least if other MLM opportunities are not present. In what follows I analyse the case of a monopoly MLM firm, using the case where commission rates vary by level (as in Eq 1A).

#### 3.3.1. Distributors compete under a fixed price

Even if the product is manufactured by a monopoly MLM firm, the existence of many distributors (some at the same level as others) means that they compete with each other. Therefore, for now I assume a fixed price which is a result of perfect competition between distributors. The market price will be determined by the marginal distributor–i.e., the distributor at the lowest level. In equilibrium, this distributor has zero economic profits. In other words, his revenues from selling the product have to equal his outside option, which I assume to be the minimum wage. Note that this distributor has no downline distributors, by definition. The zero profit condition is given in Eq 16:

$$[P-c-I\alpha ]Q_{I}(t)=wL$$ (16)

Therefore, the equilibrium price is given by:

$$P=\frac{wL}{Q}+c+I\alpha$$ (17)

Essentially, market price is positively affected by the wholesale price, upline fees, and the attractiveness of the outside offer. It is also positively affected by the number of MLM levels in the firm. Finally, it is negatively associated with the quantity sold, or more exactly, with the ability of the distributor to sell large quantities.

The price offered by this marginal distributor, which is the distributor at level I, includes fees paid to upline distributors. This means that upline distributors are able to offer lower prices, since they pay lower upline fees. The reason they do not choose to do so is that in our model, the quantity sold depends on time devoted to sales. These distributors cannot increase the quantity sold without increasing *t*. Therefore, they adopt the price of the distributor at level *I*. If we compare the results of this model to the fully competitive case (Section 3.B), we see the price is the same in both models (The price formula in Eq 10 equals the price of the competitive case (C+m) after plugging in m using Eq 3). In other words, this model is no different from the competitive model. This result is due to the competition between distributors in this model, and the limits to how much each distributor can sell (i.e., the fact that his sales depend on *t*). Since this case of a perceived monopoly is in effect a competitive market and thus does not provide new insights, let us move to another case of monopoly, where both the firm and each distributor have market power.

#### 3.3.2. Allowing market power within the MLM firm

Here I assume that each distributor can sell as much as he wants (*Q* doesn’t depend on *t*). This will be the case if, for example, internet sales are a possibility. This case gives an advantage to upper-level distributors, as they have fewer fees to pay and can therefore charge lower prices without compromising their profits compared to downline distributors. This is why I now analyse the decision making of the uppermost distributor. I assume that in addition to the monopoly power of the MLM firm, each distributor has market power–the ability to set his own price. The maximization problem of the uppermost distributor (where *i = 1*) is given by:

$$\pi_{1}=[P-C]Q_{1}(P)+\alpha \sum_{j=2}^{I}\left[Q_{j}(P)\right]$$ (18)

The first-order condition with respect to *P* generates the following condition:

$$P=C-\frac{Q_{1}}{{dQ}_{1}/dP}-\alpha \{\sum_{j=2}^{I}\left[{dQ}_{j}/dp]\}/(dQ_{1}/dP\right)$$ (19)

In other words, price is determined by production costs, price elasticity of demand and an MLM-specific expression (the first, second and third terms of the right-hand side of the equation, respectively). The first two terms are similar to the standard monopoly formula–price margin over cost is inversely proportional to the elasticity of demand. The third term on the right is an additional component which is related to the MLM structure. Here, an increase in *α*, the commission fee, reduces the monopoly price. This happens because the price charged by the uppermost distributor influences the prices charged by his downlines. This gives him an incentive to lower his price, thus allowing lower prices and higher sales by his downline distributors who will pay him fees. This price reduction, in turn, depends on the number of levels in the firm and on the elasticity of demand. In other words, a monopoly MLM firm will charge a lower price compared to a standard monopoly.

## 4. Additional concerns

This section deals with additional concerns regarding the validity of the model and sketches directions for future research. Specifically, the model abstracts from four important business aspects: risk, self-purchase, time discounting and bounded rationality. I will discuss each aspect independently and show that the model is conservative in nature, i.e. it provides an upper-bound estimate regarding the number of levels the MLM firm will have in equilibrium.

The business opportunity of becoming an MLM distributor doesn’t come without risk. The to-be distributor has to invest time and money in order to start selling products. This is especially so for people without background in marketing who want to become distributors. While risk is indeed a potentially important factor, adding it to the model shouldn’t necessarily change the model in a significant way. One can add a probability of failure into the profit equation and thus add uncertainty regarding earnings and profits. To the extent that becoming a distributor is riskier than the outside option, adding risk into the model would increase the profits needed to persuade the distributor not to take the outside option. Therefore, the number of distributors, and hence the number of levels, is projected to be lower when risk is incorporated into the model. In that sense the model’s estimates for the number of levels in the MLM firm is an upper bound, since it does not take into account risk and uncertainty.

Some MLM firms force their distributors to buy the product for self-purchase. While the model abstracts away from such a condition, adding it wouldn’t affect the results much. Forcing the distributors to buy for self-purchase would lower their earnings and thus make this business activity less appealing. This condition, all else equal, will also work to lower the number of distributors and the number of levels.

Another simplification of the model is its static nature. All earnings are received simultaneously and the time dimension is not discussed. In reality, though, earnings are received over several periods. Moreover, earnings by downline distributors will probably take more time to materialize compared to earnings from the distributor’s own sales. An easy fix to this issue is to introduce a discount factor to the earnings which are the results of downline sales. This discount factor will reduce the incentive to recruit downline distributors and will therefore result in fewer levels. In other words, dealing with time discounting will also lower the number of levels the model predicts.

Finally, the model assumes rational agents working to maximize their earnings potential. To the extent such an assumption is incorrect the model will be able to deliver realistic estimates of actual MLM firm structure only in the long run. If agents are indeed only partially rational, they might be more easily persuaded into exploring the MLM business opportunity. In that case the MLM might be comprised of more levels and include more distributors, at least in the short run (see Section 3.2). However, keeping the MLM firm at these elevated levels will mean continually recruiting additional entrants, to replace past distributors who weren’t successful (or recruiting irrational agents who do not learn over time). The long-run equilibrium will collapse back to our estimates since the number of boundedly rational agents who enter the business despite lack of earnings potential will run out with time. It is also worth noting that such boundedly rational agents may dedicate more time to recruitment at the expense of direct sales, leading to lower income and lower fees to upline distributors. In other words, we will end up with more distributors, more levels, but not necessarily higher profits.

## 5. Conclusions and policy implications

This paper presents an economic model which analyses the operation of an MLM firm in a competitive market. The model analyses the distributor level and assumes that distributors seek to maximize their earnings, and that they may choose how to divide their time between direct sales and the recruitment of downline distributors. The model focuses on the lowest-level distributor, whose behaviour recursively affects the behaviour of upline distributors. Finally, I analyse the case of an MLM firm with market power at both the firm and distributor levels.

The model provides us with several interesting observations. First, it predicts that MLM firms will have only a small number of levels in equilibrium. An upper-bound estimate suggests that a typical MLM firm will include up to nine levels, and possibly no more than six levels. This result is very far from the promises of many of these firms, which boast of their “multi-level” structures. The empirical evidence provided by the literature seem to be in line with this prediction. This small number of levels means that the business opportunity provided by becoming an MLM distributor is not as appealing as it might sound. In fact, the median distributor should expect most of his earnings to come from direct sales and not from commissions from his downline distributors. The second version of our model reaches similar conclusions, but instead of a cap on the number of levels it puts a cap on the number of distributors. I provide a simulation for the first version of the model as well as motivating examples based on actual data for both versions of the model.

Second, the model explains why MLM firms tend to concentrate in industries with high marketing costs: distributors engage in marketing activity, and if marketing cost in non-MLM firms is small the MLM firm will find it hard to compete. The model also explains how marketing costs are linked to the size of commission fees, and the fact that MLM firms seem to offer fees similar in magnitude. Where spending on marketing is low, it is not possible to charge large commission fees. This constraint caps the size of commission fees.

At the individual level, the model shows that individuals with a small outside offer will find the opportunity to become an MLM distributor more appealing. Such individuals are likely to include poorly educated populations in developed countries, as well as the majority of the population in developing countries. This explains the fast penetration rates of MLM firms in developing countries. Since these populations may be less informed about the potential weaknesses of the MLM business model, our findings highlight the need for government regulation to ensure that these distributors are not taken advantage of.

The model also analyses cases of monopoly, first of the MLM firm and then at both the firm and distributor levels. A monopoly MLM firm appears to be no different to an MLM firm operating in a competitive market, since distributors compete against each other. For the second case, that of a monopoly distributor, we can reach the surprising conclusion that MLM monopolies might charge *lower* prices compared to standard monopolies. While these prices are still higher than those found in competitive markets, this result suggests that monopoly MLM firms pose a smaller threat to consumer welfare compared to standard monopolies. From a policy perspective, it appears that MLM firms pose a greater threat to their distributors’ welfare than to consumer welfare.

## Supporting information

S1 Appendix A non-recursive model. (DOCX)
