# Supplementary material for: An economic model of multi-level marketing
Source: PLoS One. 2021 Jul 20;16(7):e0253700. doi: 10.1371/journal.pone.0253700 (PMC8291665; doi:10.1371/journal.pone.0253700)
Supplement: S1 Appendix — (DOCX) [file pone.0253700.s001.docx]

**S1 Appendix. A non-recursive model**

This model is similar to the one presented in the paper, in both substance and notation. The one important difference is in the assumption regarding the possible number of levels. Here I assume an infinite number of levels while in the baseline model I assumed a finite number of levels. Assuming infinite levels allows us to look on a representative distributor at level *i*, without specifying the exact level.

The earnings of a distributor at level i, denoted by$\pi_{i}$, are given in Equation A1:

$$\left( A1 \right)\pi_{i}=\left[ P-C-i\alpha\right]Q_{i}(t)+\alpha\sum_{j=i+1}^{I} Q_{j}\left( t_{j,}1-t \right)$$

Maximizing the profits of distributor i with respect to t produces the following first-order condition:

$$(A2) \left[ P-C-i\alpha\right]\left( \frac{dQ_{i}}{dt} \right)=\alpha\sum\frac{dQj}{dt}$$

In other words, the marginal revenue from dedicating an additional unit of time to sales (i.e., the left-hand side of Equation A2) equals the marginal revenue from dedicating an additional unit of time to recruitment (the right-hand side of the equation).

One important result of this model is that the fraction of time devoted to recruitment, namely 1-t, will dictate the size of the firm, i.e., total sales. In order to find 1-t (and t) we need to add further assumptions. Let us assume the following Cobb-Douglas production functions: $Qi=t^{0.5}$ and $Qj={(1-t)}^{0.5}$. Plugging in these production functions to Equation A1 and maximizing with respect to t yields the following condition:

$$\left( A3 \right) t=\frac{\left[ P-C-i\alpha\right]^{2}}{\left[ P-C-i\alpha\right]^{2}+I^{2}\alpha^{2}}$$

In other words, the share of time devoted to actual sales (t) is positively affected by the market price (P), but negatively affected by the distributor’s level (or position) in the organization (i), the total number of levels (I), the product cost (C), and the commission fee ($\alpha$). The reverse is true for time spent on recruitment (1-t). Time spent on recruitment is positively affected by the commission fee, product cost, total number of levels, and position in the organization, and negatively by the market price. With respect to the distributor’s position in the organization, this means that the lower the distributor’s position the more time he spends on recruitment, and the less time he spends on sales.

The result regarding the effect of i, position in the MLM structure, on time spent recruiting is striking. One might expect that lower-level distributors would dedicate more time to direct sales. However, commission fees mean that distributors at lower levels earn less from direct sales than higher-level distributors, giving them a greater incentive to spend time recruiting. Notice that there is an implicit assumption that recruitment efforts yield the same results for upper- and lower-level distributors.

The other mechanisms of the model are fairly intuitive. A higher market price increases direct sales while a higher product cost reduces them; commission fees induce greater recruitment efforts (i.e., increase 1-t); and being part of a larger organization (higher I) also increases recruitment efforts.
